# Characteristics of the IVF Cycle that Contribute to the Incidence of Mosaicism

**DOI:** 10.3390/genes11101151

**Published:** 2020-09-30

**Authors:** Lorena Rodrigo, Mónica Clemente-Císcar, Inmaculada Campos-Galindo, Vanessa Peinado, Carlos Simón, Carmen Rubio

**Affiliations:** 1 Preimplantation Genetic Testing Department, Igenomix, 46980 Valencia, Spain; inmaculada.campos@igenomix.com (I.C.-G.); vanessa.peinado@igenomix.com (V.P.); 2 Functional Genomix & Bioinformatics Lab, Igenomix, 46980 Valencia, Spain; monica.clemente@igenomix.com; 3 Department of Obstetrics and Gynecology, University of Valencia/Instituto de Investigación Sanitaria (INCLIVA), 46016 Valencia, Spain; carlos.simon@igenomix.com; 4 Department of Obstetrics and Gynecology, School of Medicine, Stanford University, Stanford, CA 94305, USA; 5 Department of Obstetrics and Gynecology, Baylor College of Medicine, Houston, TX 77030, USA; 6 Head of Scientific Advisory Board, Igenomix and Igenomix Foundation, 46980 Valencia, Spain; 7 Research & Development Department, Igenomix and Igenomix Foundation, 46980 Valencia, Spain; carmen.rubio@igenomix.com

**Keywords:** PGT-A, NGS, aneuploidy, mosaicism, segmental, vitrification, ovarian response, female age

## Abstract

Highly sensitive next-generation sequencing (NGS) platforms applied to preimplantation genetic testing for aneuploidy (PGT-A) allow the classification of mosaicism in trophectoderm biopsies. However, the incidence of mosaicism reported by these tests can be affected by a wide number of analytical, biological, and clinical factors. With the use of a proprietary algorithm for automated diagnosis of aneuploidy and mosaicism, we retrospectively analyzed a large series of 115,368 trophectoderm biopsies from 27,436 PGT-A cycles to determine whether certain biological factors and *in vitro* fertilization (IVF) practices influence the incidence of overall aneuploidy, whole uniform aneuploidy, mosaicism, and TE biopsies with only segmental aneuploidy. Older female and male patients showed higher rates of high-mosaic degree and whole uniform aneuploidies and severe oligozoospermic patients had higher rates of mosaicism and only segmental aneuploidies. Logistic regression analysis identified a positive effect of female age but a negative effect of embryo vitrification on the incidence of overall aneuploid embryos. Female age increased whole uniform aneuploidy rates but decreased only segmental aneuploidy and mosaicism, mainly low-mosaics. Conversely, higher ovarian response decreased whole uniform aneuploidy rates but increased only segmental aneuploidies. Finally, embryo vitrification decreased whole uniform aneuploidy rates but increased mosaicism, mainly low-mosaics, compared to PGT-A cycles with fresh oocytes. These results could be useful for clinician’s management of the IVF cycles.

## 1. Introduction

Current genetic analysis platforms used in preimplantation genetic testing for aneuploidy (PGT-A) are highly sensitive and, when applied to trophectoderm embryo biopsies (TE), identify mosaicism when not only euploid or aneuploid cells, but a combination of both are present in the TE biopsy. Embryonic mosaicism is mitotic in origin and is caused by improper separation and segregation of chromosomes during cell division [1]. Next-generation sequencing (NGS)-based platforms are most commonly used in PGT-A programs; however, differences in platform sensitivity and specificity, the threshold established for data interpretation and the cut-offs applied for low-level mosaicism classification affect the percentage of mosaicism reported among genetic laboratories and the number of euploid embryos deemed suitable for transfer [2]. Other factors related to biopsy technique, the number of cells biopsied, and the conditions surrounding the cell loading can also affect the results [3]. Studies by Popovic et al. [4,5] on the inner cell mass (ICM) and TE analysis from aneuploid and mosaic embryos suggest limitations in the accuracy of diagnosing mosaicism in PGT-A due to difficulty distinguishing technical bias from biological mosaicism. As a result, the use of validated NGS platforms and the development of algorithms to improve mosaicism diagnosis have become priorities for many genetic laboratories [6,7,8]. 

Ovarian stimulation protocols during the IVF cycle can influence the incidence of euploid embryos [9]. Additionally, the fertilization method [10] and conditions in the clinical IVF laboratory, such as the embryo culture media, pH, oxygen, osmolality, temperature or plastics, are linked to increased aneuploidy and mosaicism [3]. Accurate mosaicism determination describes incidences of euploid/aneuploid mosaicism from 31% at the cleavage stage to 4–5% at the blastocyst stage [11,12]. Maternal age is a major influence on aneuploidy, but primarily related to incidence of uniform aneuploidies. Isolated mosaicism may be independent of maternal age, but Rubio et al. [13] described a slight decrease in mosaicism in women over 37 compared to younger patients. The same study, however, did not observe an effect of the ovarian response and vitrification of oocytes on mosaicism rate. Finally, the use of PGT-A in couples with compromised semen quality shows higher rates of mosaic embryos with low sperm concentrations [14,15].

Since the first pregnancies reported by Greco in 2015 [16] transferring mosaic embryos after PGT-A analysis, more clinicians are considering transfer in the absence of euploid embryos. Clinical outcomes are poorest for high-mosaic compared to low-mosaic embryo transfers [17]; therefore, mosaic degree classification is critical to the decision-making process for clinicians and patients. We evaluated the distribution of euploid and aneuploid cells in a large series of 115,368 TE biopsies to assess whether the distribution was affected by characteristics of the IVF cycle, including the indication to perform a PGT-A cycle, the use of fresh or vitrified oocyte/embryos, the ovarian response, sperm concentration, and maternal and paternal ages. Aneuploidies were analyzed considering uniform aneuploidies, low- and high-degree mosaicism, and segmental aneuploidy. Logistic regression analysis identified maternal age, ovarian response, and PGT-A cycles with vitrified embryos as the factors with the most influence on aneuploidy.

## 2. Materials and Methods 

### 2.1. Study Design

This was a retrospective observational study carried out between October 2018 and December 2019 that included TE embryo biopsies from PGT-A cycles analyzed in our laboratory subsidiaries (Igenomix, Valencia, Spain). Every TE biopsy was classified as euploid or aneuploid. The aneuploidies observed in the TE biopsies were distributed among four categories: (i) only segmental aneuploidy, when only partial deletion/duplications were observed; (ii) low-mosaic, when one or two low-mosaic degree aneuploidies without additional uniform or segmental aneuploidies were observed; (iii) high-mosaic, when one or two high-mosaic degree aneuploidies or one low- and one high-mosaic degree aneuploidies without additional uniform or segmental aneuploidies were observed; or (iv) whole uniform aneuploidy, when at least one aneuploidy for a whole chromosome was observed in the TE biopsy, combined or not combined with additional segmental or mosaic aneuploidies. 

The distribution of euploid and aneuploid results within the four categories of aneuploidies (whole uniform aneuploidy, low-mosaic, high-mosaic, and only segmental) were evaluated according to the following characteristics of the IVF cycle: the indication to perform the PGT-A cycle, the origin of the embryos, ovarian response, sperm count, and maternal and paternal age. Patients with altered karyotype, cycles with females aged ≥ 45 years at embryo biopsy, as well as ovum donation cycles where excluded from analysis.

### 2.2. Study Population

The study included 115,368 day 5, 6, or 7 TE biopsies from 27,436 PGT-A cycles. Clinical indications for PGT-A were: (1) advanced maternal age, ≥37 years old (AMA; *n* = 48,174 TE biopsies); (2) repetitive implantation failure with ≥2 failures (RIF; *n* = 6742 TE biopsies); (3) recurrent pregnancy loss with ≥2 miscarriages (RPL; *n* = 5244 TE biopsies); (4) male factor infertility with impaired sperm parameters and/or increase in the incidence of aneuploid sperm (MF; *n* = 6562 TE biopsies); (5) previous aneuploid conception (PAC; *n* = 512 TE); (6) mixed causes (MIX; *n* = 2199 TE biopsies); or (7) aneuploidy screening (AS; *n* = 45,935 TE biopsies). Female age in RIF, RPL, MF, PAC, MIX, and AS indications was <37 years old. 

Information on embryo origin was available in 34,346 (29.8%) of the embryos analyzed, distributed as fresh oocyte origin (FO; *n* = 30,312 TE biopsies), vitrified oocyte origin (VO; *n* = 1649 TE biopsies), and vitrified embryo origin (VE; *n* = 2385 TE biopsies). A total of 14,092 (12.2%) TE biopsies had information regarding the ovarian response, considered as the total number of MII oocytes retrieved in the IVF cycle. These were distributed as: (i) ≤5 MII oocytes (*n* = 1677); (ii) 6–10 MII oocytes (*n* = 3302); (iii) 11–15 MII oocytes (*n* = 2646); (iv) 16–20 MII oocytes (*n* = 2373); and (v) >20 MII oocytes (*n* = 4094). The sperm count of the samples used in IVF cycles was available for 8360 (7.3%) of the TE biopsies analyzed, classified as: (i) normozoospermia with ≥15 × 10^6^ sperm/mL (N; *n* = 6428); (ii) moderate oligozoospermia between >5 × 10^6^ and <15 × 10^6^ sperm/mL (MO; *n* = 911); and (iii) severe oligozoospermia with ≤5 × 10^6^ sperm/mL (SO; *n* = 1021).

### 2.3. Next-Generation Sequencing of TE Biopsies

TE biopsies were performed on day 5, 6, or 7 of blastocyst growth. NGS analysis was conducted using an Ion ReproSeq^TM^PGS kit for 24 chromosome aneuploidy screening (Thermo Fisher Scientific, Waltham, MA, USA) and was performed on the Ion Chef^TM^ and Ion S5 System instruments (Thermo Fisher Scientific). Data were analyzed with Ion Reporter Software using the human genome build (hg19) (Thermo Fisher Scientific). An internally validated algorithm was applied for automatic mosaicism calling. Low mosaic degree was determined when the TE biopsy had 30% to <50% of aneuploidy cells while a high mosaic degree was determined by 50% to <70% aneuploid cells. TE biopsies with <30% aneuploid cells were classified as euploid and those with ≥70% were classified as uniform aneuploid [13]. TE biopsies showing another uniform aneuploidy were not reported as mosaic but as uniform aneuploid. Partial deletions/duplications were determined by >10 Mb. 

### 2.4. Statistical Analyses

Univariant analysis using pairwise comparisons between pairs of proportions with correction for multiple testing (based on Pearson’s Chi-squared test) was applied to evaluate the distribution of embryo ploidy (euploid and aneuploid) and the category of aneuploidy (whole uniform aneuploidy, only segmental aneuploidy, low-mosaic degree, or high-mosaic degree) according to the indication to perform PGT-A (AMA, RIF, RPL, MF, PAC, MIX, or AS), the ovarian response (≤5 MII oocytes; 6–10 MII oocytes; 11–15 MII oocytes; 16–20 MII oocytes; >20 MII oocytes), the classification of sperm count (N, MO, or SO) and the embryo origin (FO, VO, or VE). To evaluate the distribution of the four categories of aneuploidies according to maternal and paternal age, a pairwise comparison between means (± standard deviation, SD) with corrections for multiple testing (based on *t*-test) was used. 

Multivariant analysis was performed in 4885 TE biopsies for which all variables were present (4.4% of the informative embryos). A binary logistic regression model was applied to evaluate the variables affecting embryo ploidy and the four categories of aneuploidies. Maternal and paternal age, sperm concentration, and ovarian response defined as the number of mature oocytes retrieved (MII) were considered as quantitative variables and the embryo origin and the indication for the PGT-A were considered qualitative variables in the model, considering FO for the embryo origin and AS for the indication for the PGT-A as references. The odds ratio for each coefficient and the confidence interval at 95% were computed in the case of a significant variable to analyze the effect of each parameter. 

Statistical analysis was performed using R Free software (R Foundation for Statistical Computing, Vienna, Austria) and GraphPad InStat v3.10 for Windows (GraphPad Software, Inc., San Diego, CA, USA). Differences were considered statistically significant when *p* < 0.05, highly significant when *p* < 0.01, and extremely significant when *p* < 0.001.

## 3. Results

We retrospectively analyzed 115,368 TE biopsies and 111,860 were informative. From the informative TE biopsies, 46.1% were euploid and 53.9% were aneuploid, mainly due to whole uniform aneuploidies (42.6%) followed by mosaicism (6.2%) and TE biopsies with only segmental aneuploidies (5.1%) (Table 1). When analyzing the data per day of biopsy from day-5 to day-7, the percentage of euploid TE biopsies decreased significantly (49.4% in day 5; 42.1% in day 6; 35.7% in day 7; *p* < 0.0001) and the percentage of aneuploid embryos increased significantly (50.6% in day 5; 57.9% in day 6; 64.3% in day 7; *p* < 0.0001). The incidence of TE biopsies with whole uniform aneuploidy increased significantly from day-5 to day-7 (39.5%, 46.5% and 52.8 in day-5, day-6 and day-7, respectively; *p* < 0.0001). No significant differences in the percentage of TE biopsies with mosaicism or only segmental aneuploidies were observed in different biopsy day. 

### 3.1. Univariant Analysis

#### 3.1.1. Maternal and Paternal Age 

**Figure 1:**
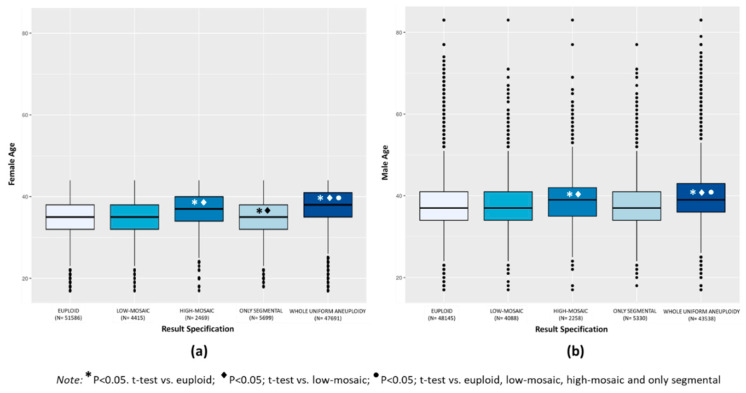
Box plot distribution of mean maternal age (**a**) and mean paternal age (**b**) expressed in years in the results categories of euploid, low-mosaic, high-mosaic, only segmental, and whole uniform aneuploid TE biopsies.

Euploid TE biopsies showed significantly lower mean female and male age compared to aneuploid TE biopsies (34.8 ± 4.2 vs. 37.0 ± 4.4 for female, and 37.6 ± 6.0 vs. 39.4 ± 6.2 for male, respectively; *p* < 0.0001). When considering the categories of aneuploidies, mean female age was similar in the groups of euploid (34.8 ± 4.2) and low-mosaic degree (34.9 ± 4.2) TE biopsies and both were slightly but significantly higher compared to only segmental aneuploidy TE biopsies (34.6 ± 2.2; *p* < 0.001) and significantly lower compared to the high-mosaic degree (36.5 ± 4.3) and whole uniform aneuploid TE biopsies (37.5 ± 4.2) (*p* < 0.0001) (Figure 1a). Mean paternal age was similar in the euploid (37.6 ± 6.0), low-mosaic degree (37.8 ± 6.0), and only segmental aneuploidy (37.5 ± 6.0) TE biopsies and these were significantly lower compared to the groups of high-mosaic degree (39.0 ± 6.1) and whole uniform aneuploid (39.8 ± 6.1) TE biopsies (*p* < 0.0001) (Figure 1b). In summary, whole uniform aneuploidy had the highest mean maternal and paternal age, significantly increased compared to the other categories of aneuploid TE biopsies (*p* < 0.0001).

#### 3.1.2. Ovarian Response

**Figure 2:**
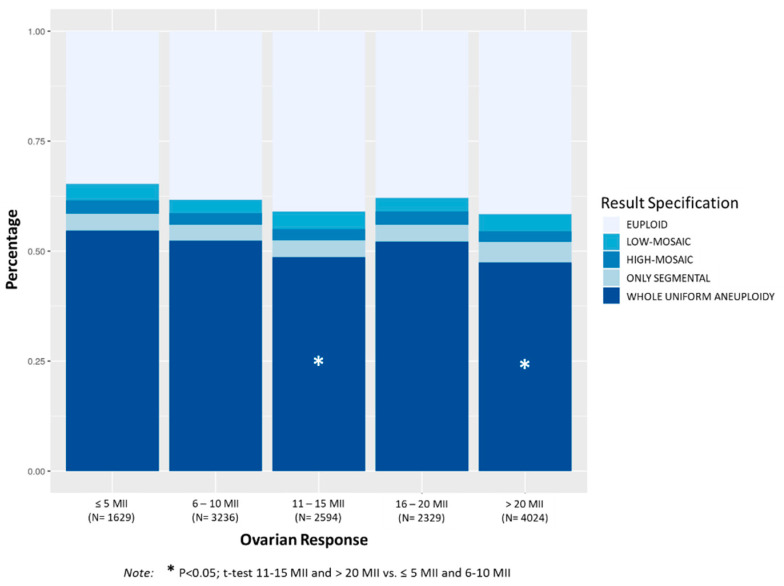
Distribution of chromosomal abnormalities in TE biopsies (euploid, low-mosaic, high-mosaic, only segmental, and whole uniform aneuploidy) according to categories of ovarian response considering the number of mature oocytes retrieved (≤5 MII oocytes; 6–10 MII oocytes; 11–15 MII oocytes; 16–20 MII oocytes; >20 MII oocytes). The mean number of embryos biopsied (SD) per case was 2.6 (1.9) for ≤5 MII oocytes category, 3.4 (2.1) for 6–10 MII, 4.5 (2.9) for 11–15 MII, 4.8 (2.7) for 16–20 MII and 6.5 (3.9) for >20 MII oocytes category.

Figure 2 shows the distribution of aneuploidies in TE biopsies according to the number of mature oocytes (MII) retrieved. The percentage of aneuploid TE biopsies decreased progressively as the number of MII oocytes increased. The percentage of whole uniform aneuploidies was significantly higher in patients with less than 11 MII oocytes compared to patients with more than 11 MII oocytes (*p* < 0.01). No significant differences in the percentage of TE biopsies with mosaicism or only segmental aneuploidies were observed within the ovarian response. 

Mean maternal age was significantly higher in the group with ≤5 MII (38.9 ± 2.7) compared to the other groups (38.1 ± 3.3 in 6–10 MII, 37.6 ± 3.5 in 11–15 MII, 38.0 ± 3.4 in 16–20 MII, 37.4 ± 3.8 in >20 MII; *p* < 0.0001). The group with 6–10 MII compared to the group with 16–20 MI, and the group with 11–15 MII compared to the group with >20 MII showed similar mean maternal ages. 

#### 3.1.3. Classification of Sperm Count 

**Figure 3:**
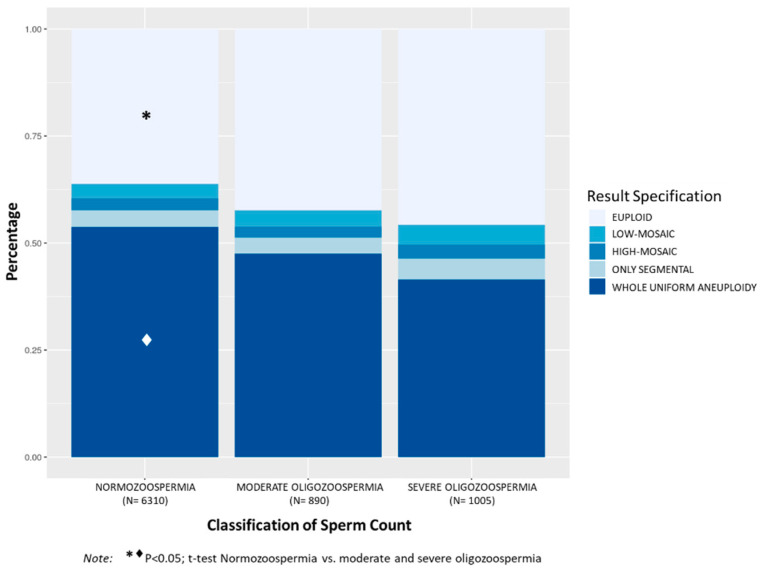
Distribution of the percentages of chromosomal abnormalities in TE biopsies (euploid, low-mosaic, high-mosaic, only segmental, and whole uniform aneuploidy) according to the classification of sperm count (normozoospermia, with ≥15 × 10^6^ sperm/mL; moderate oligozoospermia, between >5 × 10^6^ and <15 × 10^6^ sperm/mL; severe oligozoospermia, with ≤5 × 10^6^ sperm/mL). The mean number of embryos biopsied (SD) per case was 4.6 (3.2) for N category, 5.2 (4.1) for MO and 5.1 (3.7) for SO category.

Analysis of TE biopsies considering patient sperm count showed significantly higher incidence of aneuploid embryos in patients with N (63.8%) compared to patients with MO (57.6%, *p* < 0.001) and SO (54.2%, *p* < 0.0001) (Figure 3). Mean maternal age was also significantly higher in the N group (38.8 ± 3.1) compared to MO (37.4 ± 3.5) and SO (35.9 ± 3.8) (*p* < 0.001, among groups). The N group showed significantly higher incidence of embryos with whole uniform aneuploidy (53.8%) compared to MO (47.5%) and SO (41.5%) groups, *p* < 0.001. Although the percentage of embryos with mosaicism (6.1% in N; 6.4% in MO; 7.9% in SO) and only segmental aneuploidy (3.9% in N; 3.7% in MO; 4.9% in SO) was slightly increased in patients with SO, no statistical differences were observed among groups. However, the incidence of embryos with aneuploidies due to mosaicism or only segmental instead of uniform aneuploidy was significantly higher in the SO compared to the N group (12.7% vs. 10.0%, respectively; *p* < 0.05). 

#### 3.1.4. Embryo Origin

**Figure 4:**
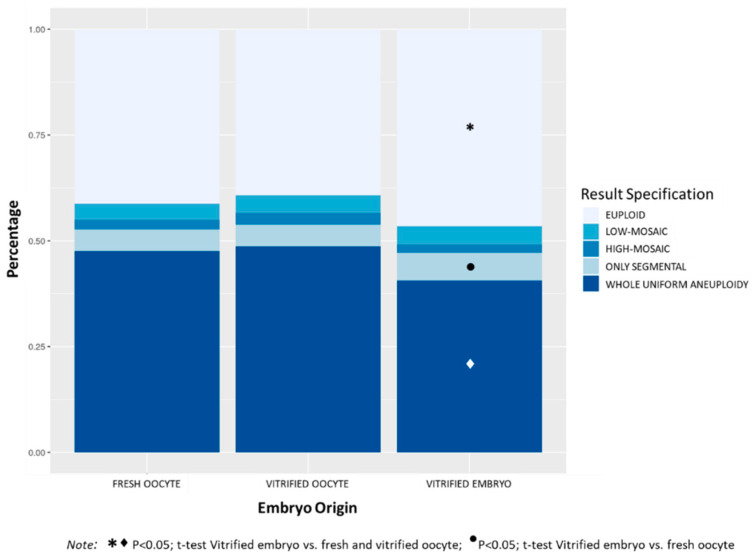
Distribution of the percentages of chromosomal abnormalities in TE biopsies (euploid, low-mosaic, high-mosaic, only segmental, and whole uniform aneuploidy) according to the embryo origin (fresh oocyte, vitrified oocyte, and vitrified embryo). The mean number of embryos biopsied (SD) per case was 4.8 (3.3) for fresh oocyte category, 4.7 (3.4) for vitrified oocyte and 4.5 (3.5) for vitrified embryo category.

Figure 4 shows the distribution of aneuploidies in TE biopsies according to embryo origin. The percentage of euploid embryos was significantly higher in the VE group (46.5%) compared to FO (41.3%) and VO (39.2%) groups, *p* < 0.0001. Comparing the four categories of aneuploidies, the VE group showed significantly lower percentage of embryos with whole uniform aneuploidy (40.6%) compared to FO (47.5%) and VO (48.7%) groups, *p* < 0.0001. The percentage of embryos with only segmental aneuploidy was significantly higher in the VE groups (6.5%) compared to the FO group (5.2%), *p* < 0.05. No differences were observed in the incidence of mosaic embryos among the three groups (6.0%, 6.9% and 6.3% in FO, VO, and VE groups respectively, NS). The VO group had the highest mean maternal age (38.7 ± 3.7), followed by the group of FO (36.6 ± 4.5), and the VE group showed the lowest maternal age (35.7 ± 4.6) (*p* < 0.0001 among groups).

#### 3.1.5. Indication for PGT-A 

**Figure 5:**
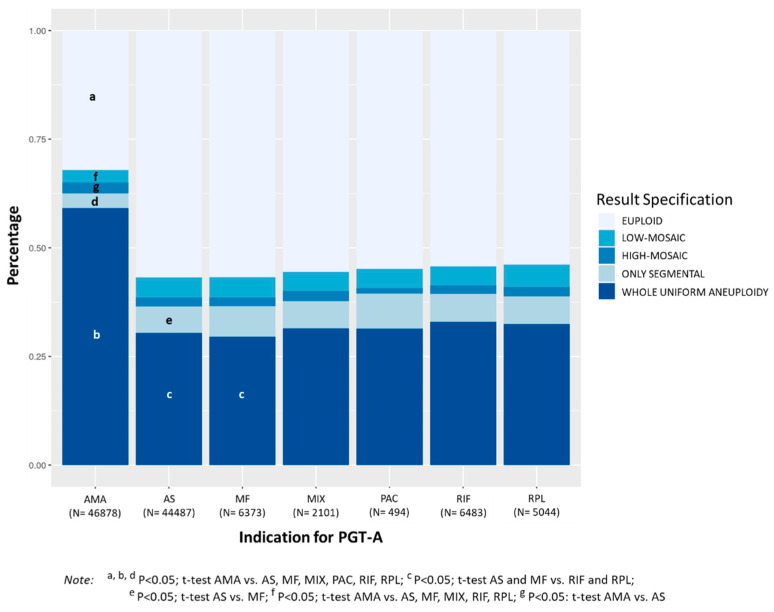
Distribution of the percentages of chromosomal abnormalities in TE biopsies (euploid, low-mosaic, high-mosaic, only segmental, and whole uniform aneuploidy) according to the indication for PGT-A (AMA: advanced maternal age ≥ 37 years; AS: aneuploidy screening; MF: male factor infertility; MIX: mixed causes; PAC: previous aneuploid conception; RIF: repetitive implantation failure; RPL: recurrent pregnancy loss). Female age was <37 years in AS, MF, MIX, PAC, RIF, and RPL groups. The mean number of embryos biopsied (SD) per case was 4.4 (3.4) for AMA indication, 6.1 (6.6) for AS, 6.0 (3.7) for MF, 5.2 (3.6) for MIX, 5.1 (2.7) for PAC, 4.8 (3.5) for RIF and 5.4 (3.8) for RPL indication.

PGT-A cycles with indication of AMA showed significantly lower percentage of euploid embryos (32.1%) compared to cycles with indication of RIF (54.2%), RPL (53.9%), MF (56.7%), PAC (54.9%), MIX (55.5%), and AS (56.8%), *p* < 0.0001 (Figure 5). Considering the four categories of aneuploidies evaluated, AMA showed significantly higher percentage of embryos with whole uniform aneuploidy (59.1%) compared to RIF (32.9%), RPL (32.5%), MF (29.6%), PAC (31.4%), MIX (31.5%), and AS (30.4%), *p* < 0.0001. Moreover, the percentage of whole uniform aneuploidy in MF and AS cycles was significantly lower than that observed in RIF and RPL cycles. 

The percentage of embryos with only segmental aneuploidy was significantly lower in AMA cycles (3.5%) compared to RIF (6.4%), RPL (6.4%), MF (7.0%), PAC (8.1%), MIX (6.3%), and AS (6.1%), *p* < 0.0001, and significantly higher in MF compared to AS (*p* < 0.05). A similar trend was observed for the overall incidence of mosaicism, which was significantly lower in AMA (5.4%) compared to RIF (6.4%, *p* < 0.01), RPL (7.3%, *p* < 0.0001), MF (6.7%, *p* < 0.001), MIX (6.7%, <0.05%) and AS (6.7%, *p* < 0.0001). The percentage of embryos with low-mosaic degree was significantly lower in AMA (2.9%) compared to RIF (4.4%), RPL (5.1%), MF (4.7%), MIX (4.4%), and AS (5.7%) (*p* < 0.0001). However, TE biopsies with high-mosaic degree were significantly increased in AMA compared to AS (2.4% vs. 2.0%, respectively; *p* < 0.001). Mean maternal age was 40.1 ± 1.7 in AMA, 33.0 ± 3.1 in RIF, 32.8 ± 3.4 in RPL, 32.6 ± 3.6 in MF, 33.5 ± 2.9 in PAC, 33.1 ± 3.2 in MIX, and 33.1 ± 3.3 in AS.

### 3.2. Multivariant Analysis

Female and male age, ovarian response, sperm count, embryo origin, and indication for PGT-A were assessed to identify which could be an independent variable affecting the overall incidence of aneuploidy and the different sub-categories (whole uniform aneuploidy, only segmental aneuploidy, low-mosaic, and high-mosaic). Logistic regression analysis showed that the percentage of aneuploid TE biopsies increased with female age (OR: 1.168, 95% CI 1.130–1.208, *p* < 0.0001), and decreased in PGT_A cycles with vitrified embryos (OR: 0.488, 95% CI 0.315–0.754, *p* < 0.01). Among the different types of aneuploidy, the percentage of whole uniform aneuploidy increased with female age (OR: 1.178, 95% CI 1.117–1.242, *p* < 0.0001). However, it decreased with higher ovarian response (OR: 0.991, 95% CI 0.985–0.998, *p* < 0.05) and in PGT-A cycles with vitrified embryos (OR: 0.416, 95% CI 0.213–0.862, *p* < 0.05). The percentage of TE biopsies with only segmental aneuploidy decreased when the female age increased (OR: 0.816, 95% CI 0.851–0.965, *p* < 0.0001) and increased when the ovarian response increased (OR: 1.011, 95% CI 0.995–1.012, *p* < 0.05). The percentage of mosaic TE biopsies decreased when female age increased (OR: 0.906, 95% CI 0.851–0.965, *p* < 0.01) and increased in PGT-A cycles with vitrified embryos (OR: 2.344, 95% CI 0.990–4.930, *p* < 0.05). Finally, analysis of the two grades of mosaicism revealed that the percentage of embryos with low-mosaic degree decreased with higher female age (OR: 0.884, 95% CI 0.817–0.958, *p* < 0.01) and increased in PGT-A cycles with vitrified embryos (OR: 3.456, 95% CI 1.279–7.879, *p* < 0.01). The percentage of TE biopsies with high-mosaic degree was not affected by any of the variables evaluated.

## 4. Discussion

PGT-A results are influenced by many analytical and biological factors. Our laboratory uses a proprietary algorithm for the automated diagnosis of aneuploidy and mosaicism in TE biopsies, resulting in an objective and robust system to minimize variability in the diagnosis [8]. We have mainly observed whole uniform aneuploidies, followed by mosaic aneuploidies and only segmental aneuploidies. Interestingly, the incidence of aneuploid embryos significantly increased from day-5 to day-7 TE biopsies, due to the increase of whole uniform aneuploidy, whereas the incidence of mosaic aneuploidy and only segmental aneuploidy remained constant regardless the day of biopsy. The increased incidence of aneuploidies in day-7 TE biopsies can be related to the lower embryo quality and significantly higher female age compared to day-5 and day-6 TE biopsies. However, other factors may contribute to differences in the overall incidence of aneuploidy and the different subtypes. To assess the influence of these other factors, we retrospectively analyzed our PGT-A results to determine if certain biological factors and IVF practices have an effect on the incidence of overall aneuploidy, whole uniform aneuploidy, mosaicism, and TE biopsies with only segmental aneuploidy. Logistic regression analysis identified maternal age, ovarian response, and PGT-A cycles with vitrified embryos as the factors with the most impact on aneuploidy determination. 

Female age is one of the primary biological factors that influences infertility. The presence of meiotic errors in oocytes increases with female age [18], which hinders successful pregnancies and makes advanced maternal age the most common indication for PGT-A. We observed a significant increase in aneuploidy with higher female age, mainly due to the increase of whole uniform aneuploidies. Girardi et al. [19] recently described a predominant meiotic origin for whole-chromosome errors, which justifies the increase in the percentage of this category of aneuploidy observed in our study. However, for other types of chromosomal abnormalities, such as segmental aneuploidies and low-mosaic degree, female age showed a negative correlation. Both could be considered as mitotic in origin [1,20]. In fact, Girardi et al. [19] showed that 67.9% of the segmental aneuploidies from preimplantation embryos had a mitotic origin, confirming previous publications [21,22]. Interestingly, mean maternal and paternal age were higher in the group of TE biopsies with whole uniform aneuploidy and high-mosaic degree compared to the group of TE biopsies with low-mosaic degree and only segmental aneuploidy. Moreover, logistic regression analysis showed a significant decrease in low-mosaic degree with female age, suggesting that this decrease is the main contributor to the overall decrease in mosaicism as female age increases. 

Regarding ovarian response, we observed a significant increase in the percentage of aneuploid embryos with the decrease in the number of MII oocytes: 58.4% in PGT-A cycles with >20 MII oocytes retrieved and 65.3% with ≤5 MII oocytes. This confirms a trend described in our previous publication [13] where there was a decrease in aneuploidies in PGT-A cycles with >20 MII oocytes (41.7%) compared to ≤5 MII oocytes (47.5%) without statistical significance. In contrast, a recent publication in cycles with oocyte donation did not find an association between the proportion of aneuploid embryos and the number of MII oocytes retrieved, although the absolute number of euploid blastocysts was higher with higher oocyte number [9]. The nature of our population could explain these differences when female age is a factor. Logistic regression analysis showed that the percentage of aneuploid embryos increases with female age, but decreases with higher ovarian response, with a stronger effect of maternal age than the ovarian response. In our population, there was a correlation between female age and the ovarian response in which patients with ovarian response below 11 MII oocytes were older than those with 11 or more MII oocytes. Female age is a risk factor for decreased ovarian reserve [23], which explains the inverse effect that both variables exerted on the overall aneuploidy rates of our population. In fact, we observed that the percentage of aneuploid embryos in the group of 16–20 MII oocytes (62.1%) was similar to the group of low responders (65.3%). This finding could be explained by the fact that female age in the group of 16–20 MII was similar to the female age in the group of low responders and significantly higher than the group with more than 22 MII oocytes. Contrary to the effect of maternal age, ovarian response was positively correlated with the incidence of TE biopsies with only segmental aneuploidies. 

Vitrification of oocytes and embryos is a widespread practice in IVF laboratories, which allows multiple possibilities in managing fertility treatment. The incidence of aneuploid embryos derived from fresh or vitrified oocytes was similar in our study, confirming previous results from our group and others [13,24]. Interestingly, we observed lower incidence of aneuploid embryos when TE biopsies derived from vitrified embryos with a decrease in whole uniform aneuploidies and an increase in TE biopsies with only segmental aneuploidies. Maternal age was lower in our group of patients using vitrified embryos compared to fresh and vitrified oocytes. Lower maternal age could explain the lower incidence of aneuploid embryos, but logistic regression analysis identified embryo vitrification as an independent factor reducing the incidence of embryo aneuploidy and increasing the probability of mosaicism mainly of low-mosaic degree. 

The contribution of sperm to embryo aneuploidy is controversial. The incidence of mosaic and chaotic patterns in cleavage stage embryos from oligozoospermic and azoospermic patients ranges from 35% to 68% [25,26,27,28,29,30,31,32]. Although the incidence is lower at the blastocyst stage, two recent studies described a higher rate of mosaic blastocysts in PGT-A cycles with male factor indication compared to couples with normal sperm parameters, with the highest rate of mosaicism correlated with the severity of male infertility [14,15]. We also observed an increase in mosaicism with a decrease in sperm count, with the highest mosaicism rate in patients with severe oligozoospermia (7.9%) similar to the 7.7% described by Tarozzi’s group and 10.9–15.6% described by Kharaman’s group [14,15]. Our group of severe oligozoospermia had the highest incidence of TE biopsies with only segmental aneuploidy. Interestingly, whereas whole chromosomal aneuploidies in TE biopsies are predominantly maternally derived aneuploidies, segmental aneuploidies are mostly paternally derived [33]. Moreover, SNP data in the same study showed 76.7% of mosaic segmental aneuploidies affecting paternally derived aneuploidies. Our group previously described sperm count as the parameter primarily associated with increased risk of sperm aneuploidies [34]. We also described a clear effect of specific sperm aneuploidies on the chromosomal constitution of preimplantation embryos [32]. Moreover, the incidence of TE biopsies with only segmental aneuploidy in male factor indication to perform PGT-A was significantly increased compared to patients performing PGT-A without a specific clinical indication (aneuploidy screening—AS). These results highlight male infertility as a factor influencing the risk of mosaicism and segmental aneuploidies in preimplantation embryos. Nevertheless, neither the sperm count nor the male factor as an indication for PGT-A were identified in the logistic regression analysis as factors affecting aneuploidy rates. The low number of samples included in the logistic regression analysis for sperm parameters (only 4.4% of the informative TE biopsies) could explain the differences observed in the univariant analysis. 

## 5. Conclusions

Female age is the most influential on the overall incidence of aneuploid embryos and mosaicism, showing an inverse effect on both. While the incidence of aneuploid embryos increases, mainly due to whole uniform aneuploidies, the incidence of mosaic embryos and only segmental aneuploidies decrease with age. Other biological and IVF factors, such as the ovarian response and PGT-A cycles with vitrified embryos also affect aneuploidy incidence, with an effect contrary to female age. While the incidence of whole uniform aneuploid embryos decreases with the ovarian response and the use of vitrified embryos, the incidence of only segmental aneuploidies increases with the ovarian response, and the incidence of mosaicism increases with the use of vitrified embryos compared to fresh oocytes. Considering the two grades of mosaicism, high mosaic degree is not affected by any of the variables evaluated. However, the incidence of low-mosaic degree decreases with female age but increases in PGT-A cycles with vitrified embryos. 

## Figures and Tables

**Table 1:** NGS results of 27,436 PGT-A cycles performed in TE biopsies.

	TE Embryo Biopsy			TOTAL
	Day 5	Day 6	Day 7	
No. embryos analyzed	64,578	50,054	736	115,368
Mean female age (SD)	35.7 (4.6) ^j^	36.3 (4.3) ^k^	36.9 (4.1) ^l^	36.0 (4.5)
Mean male age (SD)	38.5 (6.3) ^m^	38.4 (6.1) ^n^	39.2 (6.6) ^o^	38.6 (6.2)
Informative embryos (%)	62,435 (96.7)	48,703 (97.3)	722 (98.1)	111,860 (97.0)
Euploid embryos (%)	30,819 (49.4) ^a^	20,509 (42.1) ^b^	258 (35.7) ^c^	51,586 (46.1)
Aneuploid embryos (%)	31,616 (50.6) ^d^	28,194 (57.9) ^e^	464 (64.3) ^f^	60,274 (53.9)
Whole uniform aneuploidy (%)	24,672 (39.5) ^g^	22,638 (46.5) ^h^	381 (52.8) ^i^	47,691 (42.6)
Only segmental aneuploidy (%)	3187 (5.1)	2474 (5.1)	38 (5.3)	5699 (5.1)
Mosaic aneuploidy (%)	3757 (6.0)	3082 (6.3)	45 (6.2)	6884 (6.2)
Low-mosaic aneuploidy (%)	2439 (3.9)	1951 (4.0)	25 (3.5)	4415 (4.0)
High-mosaic aneuploidy (%)	1318 (2.1)	1131 (2.3)	20 (2.7)	2469 (2.2)

Note: ^a-b, a-c, d-e, d-f, g-h, g-i, j-k, j-l, k-l^ *p* < 0.0001; ^b-c, e-f, h-i^ *p* < 0.001; ^n-o^ *p* < 0.01; ^m-o^ *p* < 0.05; Fisher’s Exact test and Welch’s *t* test.
